# Spatiotemporal Characteristics, Decoupling Effect and Driving Factors of Carbon Emission from Cultivated Land Utilization in Hubei Province

**DOI:** 10.3390/ijerph19159326

**Published:** 2022-07-30

**Authors:** Pengnan Xiao, Yuan Zhang, Peng Qian, Mengyao Lu, Zupeng Yu, Jie Xu, Chong Zhao, Huilin Qian

**Affiliations:** 1 College of Urban and Environmental Sciences, Central China Normal University, Wuhan 430079, China; maikedang@mails.ccnu.edu.cn; 2 School of Business Administration, Zhongnan University of Economics and Law, Wuhan 430073, China; lumengyao@stu.zuel.edu.cn (M.L.); zuelerp@stu.zuel.edu.cn (Z.Y.); 3 Department of Economics, Party School of Henan Provincial Committee of C.P.C., Zhengzhou 451464, China; qianpeng333@163.com; 4 Faculty of Resources and Environmental Science, Hubei University, Wuhan 430062, China; 201901110800086@stu.hubu.edu.cn; 5 School of Chemistry and Environmental Engineering, Wuhan Polytechnic University, Wuhan 430040, China; zhaochong426@whpu.edu.cn; 6 Faculty of Geographical Science, Beijing Normal University, Beijing 100875, China; 201911051904@mail.bnu.edu.cn

**Keywords:** cultivated land, carbon emissions, spatiotemporal characteristics, driving factor, decoupling effect, LMDI model

## Abstract

The carbon emission level and spatiotemporal characteristics in Hubei Province were estimated and studied using the Intergovernmental Panel on Climate Change (IPCC) carbon emission coefficient technique based on county data from Hubei Province from 2000 to 2020. The relationship between carbon emissions from cultivated land utilization and agricultural economic growth was examined using the Tapio decoupling index, and the factors influencing carbon emissions in Hubei Province were further examined using the Logarithmic Mean Divisia Index (LMDI model). The results demonstrate that: (1) Spatiotemporal variations in carbon emissions are evident. In terms of time, the volume of carbon emissions in Hubei Province is still substantial, and the transition to low-carbon land use is quite gradual. Geographically, the high-value region of the middle east coexists with the low-value zone of the west, with apparent regional contrasts. (2) The decoupling between carbon emissions and agricultural economic growth is becoming more and more obvious in Hubei Province. The number of counties and cities in a negative decoupling state has significantly decreased, and the majority of counties are now in a strong decoupling condition. (3) Agricultural production efficiency is the most significant driving factor for restricting carbon emission, according to the decomposition results of carbon emission driving factors based on the LMDI model. In addition, the results of sample decomposition based on topographic characteristics indicate that agricultural production efficiency is primarily responsible for the suppression of carbon emissions in flat regions. The increase in carbon emissions in hilly regions is primarily influenced by agricultural productivity. The increase in carbon emissions in mountainous regions is mostly influenced by agricultural labor intensity. This study′s finding has enlightening implications for the high-quality growth of agriculture.

## 1. Introduction

Greenhouse gas emissions caused by human factors, such as industrial production and transportation, have brought a series of problems to the harmonious development of mankind and nature [1]. All nations, including China, are facing significant issues with and challenges related to reducing the volume of carbon emissions. To reduce the scale of agricultural carbon emissions, the No. 1 central document of the State Council of China proposed a variety of measures in 2022, such as giving priority to the overall control of agricultural non-point source pollution and emphasizing the green development of agriculture and rural areas [2]. Promoting the low-carbon utilization of cultivated land resources is not only the top priority to achieve high-quality agricultural development, but is also in line with the requirement of government policies [3].

Driven by the development of agricultural mechanization and agricultural chemistry, the economic efficiency of China′s cultivated land resource utilization has been continuously improved [4], especially in the 20 years from 1998 to 2018, and the overall carbon productivity of China′s planting industry has been on the rise [5]. However, the phenomena of a low energy consumption ratio, wide range of pollution, and unqualified emissions in the utilization of cultivated land resource are still relatively common [6]. In addition, the lack of per capita agricultural resources and the lack of technical reserves for green agricultural development in China is restricting the development of low-carbon agriculture [7]. The low-carbon utilization of cultivated land resources, as an important means of coordinating ecological environment security, food security and agricultural product quality security, has become an important way to realize the benign utilization of cultivated land resources and a key guarantee for the leapfrog development of agriculture [4]. Therefore, accurately identifying the carbon emission level of cultivated land resource utilization has become an essential premise to promote the low-carbon utilization of cultivated land resources. 

At present, researchers have made a lot of explorations around the carbon emissions related to cultivated land resource utilization under environmental constraints, mainly including: carbon emission measurement methods [4,8,9,10], carbon emission effects [11,12,13,14,15,16], carbon emission performance [17,18,19,20,21], spatiotemporal characteristics of agricultural carbon emissions [22,23,24], agricultural carbon emission driving factors [25,26], cultivated land occupation and economic development [4,8,27]. This kind of literature has important theoretical value for expanding the width and depth of research topics. The study of the carbon emission of cultivated land resource use in China has been well-founded by a number of beneficial research findings on the carbon emission of land usage; however, there are still the following shortcomings: (1) while research on the carbon emissions of cultivated land resource usage is less complicated, much of the literature focuses on construction land and urban land use [28]. (2) There has been no thorough and systematic study on the spatiotemporal features of carbon emissions from the county level, and the sample region is often restricted to the province or prefecture-level cities [29]. (3) The decoupling analysis between carbon emissions and economic growth is not deep enough, and there is little comparative analysis based on the decoupling characteristics of different regions during the same period [30]. Thus, how can a verifiable carbon emission measurement system be built for the use of cultivated land resources? What are the temporal evolution characteristics, regional differences and decoupling statuses of carbon emissions in China? These are scientific questions that need to be answered urgently.

In view of this, according to the compilation of the carbon emission measurement system of cultivated land resource utilization, this paper calculates the carbon emission level in Hubei Province from 2000 to 2020, and systematically analyzes the temporal change characteristics and regional differences of the carbon emission of cultivated land resource utilization in Hubei Province. The decoupling model, which is one of the highlights of this paper, is used to thoroughly examine the decoupling relationship between carbon emissions and agricultural economic growth in Hubei Province from various stages and perspectives, and to further analyze the driving factors of carbon emissions, in order to draw pertinent research conclusions and enlightenment, and to provide guidance for decision making for realizing the harmonious use of cultivated land resources and the high-level development of agriculture.

## 2. Materials and Methods

### 2.1. Calculation Method of Carbon Emission from Cultivated Land Use

Scholars′ understanding of the scope of carbon emissions related to the use of cultivated land resources can be divided into two types [4,31]. First, some researchers believe that the carbon emissions generated in the process of using cultivated land resources only include the direct or indirect greenhouse gas carbon emission caused by human production and operation activities [32,33]. Second, others believe that the carbon emissions generated in the process of utilizing cultivated land resources should also include the carbon sink effect of cultivated land resources and outputs [34,35]. Based on the published research results, more researchers focus on the first cognition [28,36,37].

This research examines the carbon emissions caused by the usage of cultivated land in order to provide a decision-making framework and policy suggestions for the low-carbon use of these resources in Hubei Province. The input of high-carbon compounds is reduced and soil damage may be managed, which are the distinguishing features of low-carbon exploitation of cultivated land resources [38,39]. Therefore, this study holds that greenhouse gases directly or indirectly generated by agricultural production practices account for the majority of carbon emissions from the utilization of cultivated land resources [4,8,40], including: first, the chemical utilization of cultivated land resources, such as direct or indirect carbon emissions caused by the production and use of fertilizer, pesticide, agricultural film and materials [41]. Second, energy consumption, such as direct or indirect carbon emissions caused by diesel, electricity and other energy consumed in the process of agricultural production [42]. Third, cultivated land planting, such as ploughing, destroys the soil surface and soil organic carbon pool, resulting in the release of organic carbon into the atmosphere [43].

After consulting the literature, it was found that the carbon emission measurement of cultivated land resource utilization has not yet formed a method generally recognized by many scholars [4]. Based on the Intergovernmental Panel on Climate Change (IPCC) carbon emission coefficient method [21], this research calculates the carbon emissions caused by the utilization of cultivated land resources in Hubei Province, and the formula is as follows:

(1) $$C=C_{f}+C_{p}+C_{m}+C_{e}+C_{i}+C_{t}$$

where: *C* is the total carbon emission of cultivated land resource utilization (unit: ton). As can be seen in Table 1, *C_f_*, *C_p_*, *C_m_*, *C_e_*, *C_i_*, *C_t_*, respectively, represent the carbon emissions produced by chemical fertilizer, pesticide, agricultural film, agricultural machinery, irrigation and tillage (unit: ton).

### 2.2. Decoupling Relation Model

Decoupling, which describes the connection between forces that influence the economy and those that put pressure on the environment [47], was first proposed by the Organisation for Economic Co-operation and Development (OECD) and was further divided by OECD into absolute decoupling and relative decoupling [8]. Relative decoupling indicates that economic growth is greater than the rise in resource consumption, while absolute decoupling indicates that economic growth and resource consumption are stagnant or declining [8]. However, the OECD decoupling model has two obvious defects: first, it is highly sensitive to the value of the base period and the end of the period of variables, which is prone to calculation deviation [8]. Second, the definition of decoupling connection types is too broad to differentiate the precise relationships between economic development and environmental pressure [4]. In 2005, Tapio advanced the notion of “decoupling elasticity,” also known as carbon emission elasticity, based on the enhancement of the OECD decoupling model [50]. This idea relates to the relationship between the pace of economic growth and the degree of change in carbon emissions, which may more accurately depict how sensitively changing carbon emissions are to economic development [50]. Tapio categorizes the decoupling state into eight kinds based on the decoupling elasticity value (as shown in Table 2) [51]. The decoupling elasticity eliminates the difficulty of the base period selection, and can dynamically depict the decoupling connection between variables, and has more apparent benefits for studying the link between carbon emissions and economic development [4,8]. In order to examine the decoupling link between carbon emissions and agricultural economic development in Hubei Province, this article chooses the Tapio decoupling index and builds the decoupling model as follows:

(2) $$e=\frac{\Delta C/C}{\Delta G/G}$$

where: *e* refers to decoupling elasticity; *C* refers to carbon emission from cultivated land use (unit: ton); Δ*C* refers to the change of carbon emission (unit: ton); *G* refers to agricultural output value (unit: CNY); and Δ*G* refers to the increase in agricultural output value (unit: yuan).

### 2.3. Decomposition Model of Carbon Emission Drivers

Yoichi Kaya, a famous Japanese scholar, put forward the Kaya identical equation [52], which indicates the influence of factors such as population size, GDP per capita, and energy consumption per unit gross domestic product (GDP) on the amount of carbon dioxide emissions, at the IPCC international seminar for the first time. Since then, the Kaya identical equation has been applied by many scholars to examine the influencing variables of carbon emissions in various regions and industries [53,54,55,56].

Based on Kaya identical equation, this study establishes a correlation between agricultural production efficiency, industrial structure, output level, labor scale and carbon emission, and quantitatively decomposes the driving factors of carbon emission by constructing a Logarithmic Mean Divisia Index (LMDI) model [57,58]. The LMDI decomposition method, which is a widely used and better factor decomposition method [59], can overcome the defects of zero or negative data in the decomposition process and the residual error in the decomposition results. The specific expression of the LMDI model is as follows:

(3) $$C=\frac{C}{G}\times \frac{G}{G_{A\;}}\times \frac{G_{A\;}}{P}\times P$$

(4) $$\beta_{1}=\frac{C}{G}$$

(5) $$\beta_{2}=\frac{G}{G_{A\;}}$$

(6) $$\beta_{3}=\frac{G_{A\;}}{P}$$

In Formulas (3)–(6): *C* represents the total carbon emission from cultivated land use (unit: ton); *G* refers to the total output value of planting industry (unit: CNY); $G_{A}$ refers to the total output value of agriculture, forestry, animal husbandry and fishery (unit: CNY); *P* refers to the scale of agricultural labor (unit: people); *β*_1_ means agricultural production efficiency (unit: kg·CNY^−1^); *β*_2_ represents agricultural production structure (%); and *β*_3_ refers to agricultural output level (unit: CNY·person^−1^).

(7) $$C=\beta_{1}\times \beta_{2}\times \beta_{3}\times P$$

Logarithm, addition and subtraction, decomposition and other treatments are applied to Equation (7) to obtain the contribution value of each decomposition factor of carbon emission, and the expressions are as follows:

(8) $$\Delta \beta_{1}=\frac{C^{T}-C^{0}}{\ln C^{T}-\ln C^{0}}\times \left(\ln\beta_{1}^{T}-\ln\beta_{1}^{0}\right)$$

(9) $$\Delta \beta_{2}=\frac{C^{T}-C^{0}}{\ln C^{T}-\ln C^{0}}\times \left(\ln\beta_{2}^{T}-\ln\beta_{2}^{0}\right)$$

(10) $$\Delta \beta_{3}=\frac{C^{T}-C^{0}}{\ln C^{T}-\ln C^{0}}\times \left(\ln\beta_{3}^{T}-\ln\beta_{3}^{0}\right)$$

(11) $$\Delta P=\frac{C^{T}-C^{0}}{\ln C^{T}-\ln C^{0}}\times \left(\ln P^{T}-\ln P^{0}\right)$$

(12) $$\Delta C=\Delta \beta_{1}+\Delta \beta_{2}+\Delta \beta_{3}+\Delta P$$

In Formulas (8)–(12), Δ*β*_1_ is the carbon emission effect caused by agricultural production efficiency (t); Δ*β*_2_ is the carbon emission effect caused by agricultural production structure factors (t); Δ*β*_3_ refers to the carbon emission effect caused by agricultural output level factors (T); Δ*P* is the carbon emission effect caused by the agricultural labor scale factor (T); Δ*C* is the total effect of carbon emissions caused by various influencing factors (t); *C_T_*, $\beta_{1}^{T},\;\beta_{2}^{T}$, $\beta_{3}^{T}$, $P^{T}$, respectively, represent *C* and *β*_1_, *β*_2_, *β*_3_, *P* in year *T*, while *C*_0_, $\beta_{1}^{0},\;\beta_{2}^{0}$, $\beta_{3}^{0}$, $P^{0}$, respectively, represent *C* and *β*_1_, *β*_2_, *β*_3_, *P* in base period year.

## 3. Study Area and Data Source

### 3.1. Study Area

**Figure 1:**
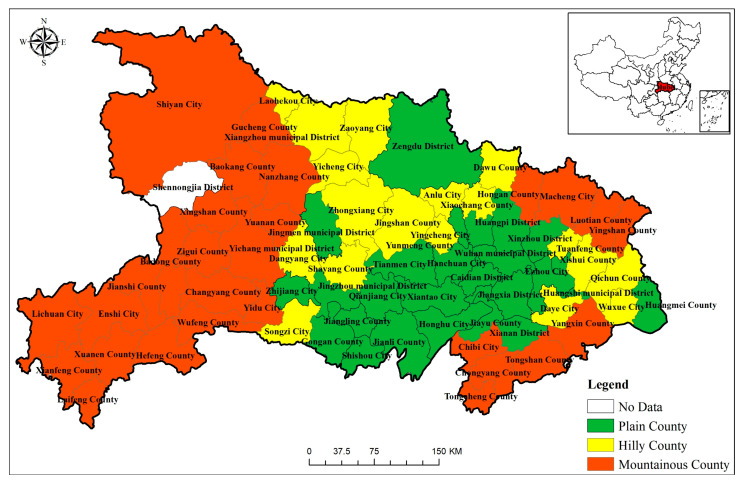
Location of study area and county type division.

In the past 30 years, Hubei Province, as a major grain producing province, has suffered a serious loss of cultivated land, and weakened the function of grain production. In addition, there are significant disparities between the east, center, and west of Hubei Province in terms of resource endowments and economic growth, and the usage of cultivated land is imbalanced. It is representative to explore the carbon emission status of cultivated land use in Hubei Province. In terms of geographical pattern, Hubei Province is characterized by large topographic relief and complex geomorphic types. It is dominated by mountains and hills. Plains (below 50 m above sea level), hills (50–200 m above sea level), and mountains (over 200 m above sea level) make up 20 percent, 24 percent, and 56 percent of the province of Hubei′s landform, respectively. Mountains are mainly distributed in western Hubei, hills are mainly distributed in northeastern, southeastern and northern Hubei and plains are in central and southern Hubei. From the perspective of an economic pattern, the county economy in Hubei Province presents a spatial distribution pattern of “low in the west and high in the east”, “high in the middle and low in the surrounding areas”, and the areas with high economic development level are primarily distributed around the districts of Wuhan and Yichang, whereas the economically underdeveloped areas are primarily located in the border areas of Hubei Province, with a concentration in the south. The specific administrative divisions of counties and cities in Hubei Province are shown in Figure 1.

### 3.2. Data Sources

The Hubei Rural Statistical Yearbook, the Hubei Province Statistical Yearbook, the statistical yearbooks of the cities and prefectures in Hubei Province, and the websites of the statistical bureaus of cities and prefectures provided the fertilizer, pesticide, agricultural film, agricultural machinery, tillage, irrigation, agricultural output value and other pertinent data used in this paper. These sources cover the years 2000–2020. Among them (Table 3), the chemical fertilizer shall be subject to the net amount of agricultural chemical fertilizer. The input amount of pesticides and agricultural plastic films shall be subject to the actual use amount in the current year. The total power of agricultural machinery shall be subject to the year-end ownership of agricultural machinery. Ploughing is replaced by the sown area of crops in that year. Irrigation is subject to the effective irrigation area in the current year. To avoid the influence of pricing considerations, the output value data are deflated and the nominal output value of each year is transformed to the actual production value estimated at the comparable price of 2000 year. 

Because the statistical data of Shenlongjia district for many years cannot be obtained, it was excluded from the study area. Some counties under the jurisdiction of Suizhou, Shiyan and Ezhou were discovered to be lacking statistical data on pesticides and agricultural films for a number of years during the process of data gathering. We amalgamated the county administrative regions under Suizhou, Shiyan and Ezhou in order to preserve the integrity and continuity of data during the study period. This study selected 72 counties (or cities) as the research object. In this paper, further considering terrain factors and classification standards [60], all counties in Hubei Province are divided into three types: plain County, hilly county and mountainous county (as shown in Figure 1). It should be mentioned that the division result does not conflict with the result of the administrative region consolidation.

## 4. Results

### 4.1. Spatiotemporal Characteristics of Carbon Emissions from Cultivated Land Use

#### 4.1.1. Time Series Change of Carbon Emissions

**Figure 2:**
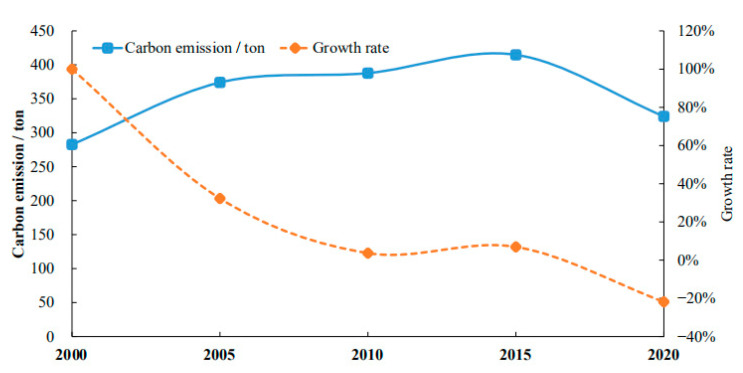
Time series change of total amount and growth rate of carbon emission.

The features of the temporal change in carbon emission and growth rate of cultivated land resource utilization in Hubei Province are obtainable (as shown in Figure 2). The results show that during the period 2000–2015, the carbon emission from cultivated land use in Hubei Province grew continuously, with an average annual increment of 10.4 tons and an average annual growth rate of 5%. Among these, the growth rate of carbon emission during the period 2000–2005 was the fastest, and the total carbon emission increased from 282.8 tons in 2000 to 374.12 tons in 2005. This can be attributed to the increase in the input of chemical products and mechanical equipment in agricultural production, which leads to the increase in the input of agricultural materials such as fertilizer, pesticide, agricultural film and agricultural machinery or the degree of soil damage, thus causing the increase in carbon emissions from the use of cultivated land resources. However, in general, the growth rate of carbon emissions from cultivated land resource utilization continued to decline from 2000 to 2010, and especially after 2015, it entered a negative growth stage. The total carbon emission in Hubei Province decreased from 414.83 tons in 2015 to 324.24 tons in 2020, a decrease of 21.84% compared with 2015. This indicates that the carbon emission from the use of cultivated land resources in Hubei Province was effectively controlled, which may benefit from the continuous practice of the concept of low-carbon green development in the field of agricultural production since the 18th National Congress of Communist Party of China (CPC). The use of cultivated land resources is changing from high carbon to low carbon.

**Figure 3:**
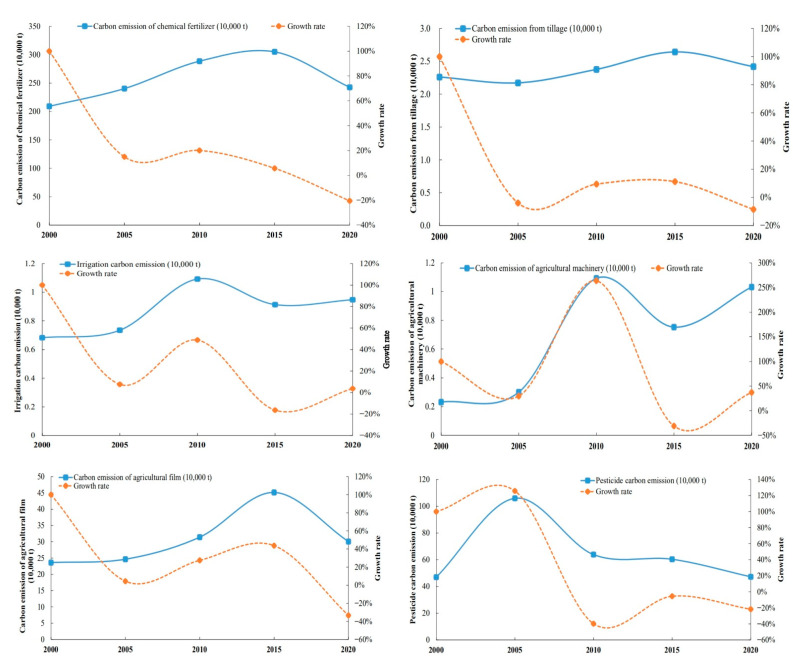
Time series change of carbon emission magnitude growth of different carbon sources.

In different historical stages or periods, due to the impact of agricultural production structure, the production mode, scientific and technological level and other factors, the different carbon source emissions and growth rate of cultivated land resource utilization show different fluctuation characteristics. Figure 3 shows that during the period 2000–2020, as the most important source of carbon emissions, the carbon emissions caused by agricultural fertilizers generally experienced a change from rising to falling, with the highest peak of 3,050,200 tons (2015), showing a downward trend from 2015 onwards. In 2020, the carbon emissions caused by the use of agricultural machinery accounted for approximately 0.32%. From 2000 to 2020, the carbon emissions from the use of agricultural machinery generally showed the fluctuation characteristics of “decline rise decline rise”, which could be attributed to the fluctuation of the use scale and efficiency of agricultural machinery caused by the change of agricultural machinery extension policies and agricultural production modes in different periods. For instance, the rate of agricultural mechanization increased after China started to apply its subsidy scheme for the acquisition of agricultural machinery and tools in 2004 [61]. During the utilization of cultivated land resources, the carbon emissions caused by ploughing accounted for 0.75%, and its carbon emissions increased from 22,600 tons in 2000 to 24,200 tons in 2020, with an average annual growth rate of only 0.35%. The overall fluctuation range was small, indicating that the overall state of the ploughing area was relatively stable during this period.

Figure 3 demonstrates that using agricultural plastic film results in 9.27 percent of all carbon emissions (2020 year). In general, agricultural film-related carbon emissions increased from 2000 to 2015, peaking at 451,300 tons in 2015. This tendency may be linked to the growing usage of agricultural film to assure increased grain output and efficiency. Since 2016, the carbon emission caused by agricultural film has shown a downward trend, which may be due to the improvement of the utilization of agricultural film and the promotion and application of low-carbon planting technology, which have reduced the use of agricultural plastic film. Since 2000, the carbon emissions caused by irrigation have shown a trend of “rising-falling-rising”, with an average annual growth rate of about 1.94%, which to some extent indicates that the continuous improvement of water conservancy facilities has led to the continuous increase in effective irrigation area. In 2020, the carbon emissions caused by pesticide input in the process of farmland utilization in Hubei province accounted for approximately 14.58%. From 2000 to 2015, carbon emissions generally showed a “rise–decline” trend, i.e., from 2005 to the trend of continuous decline, from the highest peak of 1,059,700 tons (2005 year) to 472,600 tons (2020 year), indicating that the use of pesticides, in the use of cultivated land resources, continued to decrease, which may be related to the pesticide reduction production policy in recent years.

In general, there are great differences in the farming system, planting structure and natural conditions in the different counties and regions of Hubei Province, which affect the intensive level and utilization mode of cultivated land resources. It may also be that the various agricultural materials invested in the utilization of cultivated land resources and the degree of soil damage are different, which leads to the difference of carbon emission sources in various regions.

#### 4.1.2. Spatial Variation of Carbon Emissions

**Figure 4:**
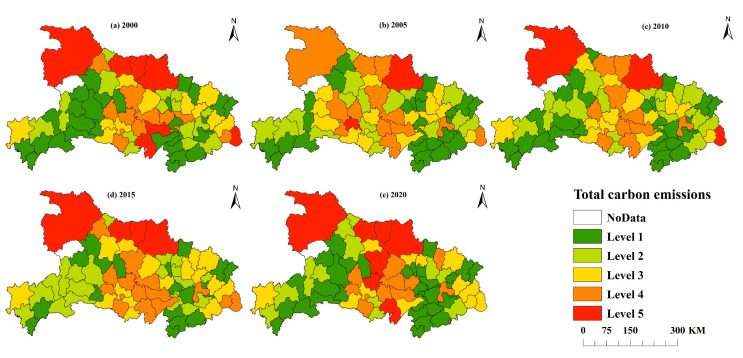
Spatial pattern of carbon emissions in Hubei Province (2000–2020).

**Figure 5:**
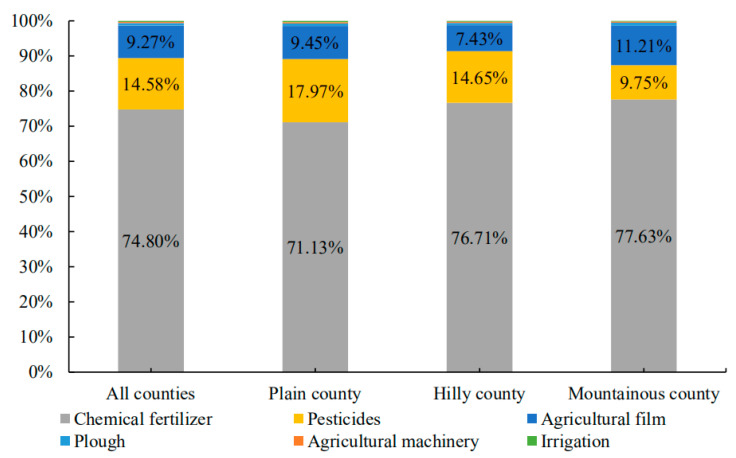
Topographic differences of different carbon emission sources.

Additionally, this paper also analyzed the spatial pattern of carbon emissions (Figure 4). The natural discontinuity approach in ArcGIS 10.7 was used to split the carbon emissions into five levels so that the variations in carbon emissions between counties may be more easily and intuitively compared. The levels with the lowest and largest carbon emissions are 1 and 5, respectively. The deeper the hue on the map, the bigger the carbon emissions at that level. From the county level, from 2000 to 2020, the number of Level 1 carbon emission counties was 29, 26, 28, 19 and 32, respectively, showing a change trend of “decrease increase decrease increase”. From the regional distribution of the number of Level 5 carbon emission counties, from 2000 to 2020, the number was 7, 2, 3, 4 and 7, respectively, showing a “decrease increase” trend. In combination with Figure 5, the changes of counties and regions included in each carbon emission level from 2000 to 2020 show that Shiyan, Suizhou and other places have long had high carbon emissions, agricultural output is limited by mountain terrain conditions, and the proportion of carbon emissions from carbon sources such as agricultural machinery and irrigation is high [59]. In 2020, the carbon emissions in Jianghan Plain will reach a high level, becoming the main source of carbon emissions from the utilization of cultivated land resources in recent years, and the inter county differences in carbon emissions tend to expand. It may be that the region is dominated by planting industry, the intensive use of cultivated land resources is high, and high-carbon production factors such as chemical fertilizer, agricultural film and pesticide are invested more [59], resulting in the high carbon emissions of cultivated land resources. At the same time, the total carbon emission has changed from “northern Hubei > central Hubei > eastern Hubei > western Hubei” in 2000 to “northwest Hubei > central Hubei > eastern Hubei > southwest Hubei” in 2020.

#### 4.1.3. Topographic Differences in the Proportion of Emissions Per Carbon Source

Additionally, as shown in Figure 5, there are variations in the proportion of different carbon sources in different regions of cultivated land resource utilization in Hubei Province. At the provincial level, chemical fertilizer, pesticide and agricultural film accounted for the highest proportion, reaching 74.80%, 14.58% and 9.27%, respectively. Plain areas developed commercial crops and are comparatively more reliant on pesticides, leading to a relatively high share of pesticide-related carbon emissions across the province. The hilly area is an important grain production area in Hubei Province. In order to ensure the continuous improvement of grain output, the use of chemical fertilizer is relatively high, resulting in a relatively high proportion of carbon emissions caused by chemical fertilizer in the region in the province. Mountainous areas are often dry and cold. In order to improve the low temperature and ensure soil moisture, the use of agricultural film is high, resulting in a relatively high proportion of carbon emissions caused by agricultural film in the region.

### 4.2. Spatiotemporal Characteristics of Carbon Emission Intensity

#### 4.2.1. Time Series Change of Carbon Emission Intensity

**Figure 6:**
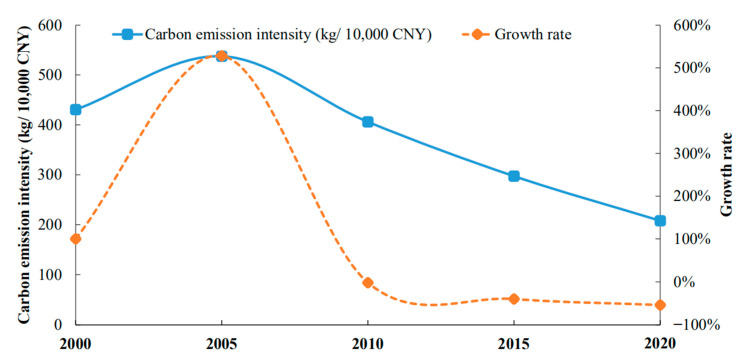
Time series change of carbon emission intensity and growth rate.

The gross agricultural product (here referring to the planting industry) mostly reflects the output of cultivated land resource utilization; hence, the carbon emission intensity may be represented as the carbon emission per unit of GDP of planting industry. The carbon emission intensity can objectively depict the degree of low-carbon cultivated land use in a particular period or place and can better assess the changes in time and geography since this indicator is unaffected by the overall resource base. Figure 6 shows that Hubei Province′s carbon emission intensity tends to shrink, falling from 430.54 kg/CNY 10,000 in 2000 to 207.85 kg/CNY 10,000 in 2020, a drop of over 52 percent. This is directly tied to advancements in agricultural technology, agricultural production methods and government initiatives to support the growth of low-carbon, environmentally friendly agriculture. The fall rate of carbon emission intensity of cultivated land resource consumption tends to slow down as a result of the marginal decreasing impact, which also indicates that the decline space of carbon emission intensity is getting smaller. 

#### 4.2.2. Spatial Variation of Carbon Emission Intensity

**Figure 7:**
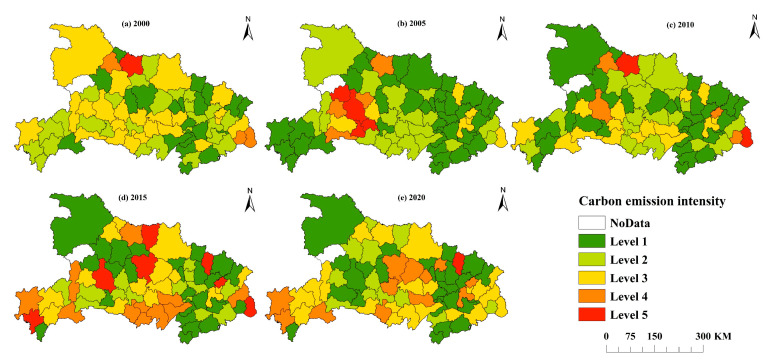
Spatial distribution of carbon emission intensity in Hubei Province (2000–2020).

This study employs the natural discontinuity approach in ArcGIS 10.7 to split the intensity of carbon emissions into five levels, with Level 1 having the lowest intensity and Level 5 having the greatest, as illustrated in Figure 7. Overall, in the 20 years from 2000 to 2020, the number of Level 5 carbon emission intensity counties and districts decreased from 7 to 1, and the number of Level 4 carbon emission intensity counties and districts decreased from 4 to 0. At the county perspective, there were 20 Level 1 carbon emission intensity counties in 2000 and 27 Level 1 carbon emission intensity counties in 2020, accounting for 77.42% of the total. The number of counties with Level 5 carbon emission intensity decreased from 1 to 0, and the number of counties with Level 4 carbon emission intensity decreased from 4 to 0. This demonstrates that the low-carbon content of cultivated land resources significantly improved in the majority of counties in Hubei Province, and the difference between counties generally tends to reduce. From the regional perspective, there was a large gap in the carbon emission intensity of different counties in Hubei Province in 2000. The carbon emission showed the characteristics of “northern Hubei > southern Hubei > western Hubei > eastern Hubei”. In Hubei Province, the difference in carbon emission intensity across the various counties tends to narrow in 2020, with the carbon emission intensity typically being low in the south and high in the north. In this regard, adjusting measures to local conditions and implementing differentiated policies will be an important idea and direction for the low-carbon emission of cultivated land resource utilization in Hubei Province.

#### 4.2.3. Topographic Differences in Carbon Emission Intensity

**Figure 8:**
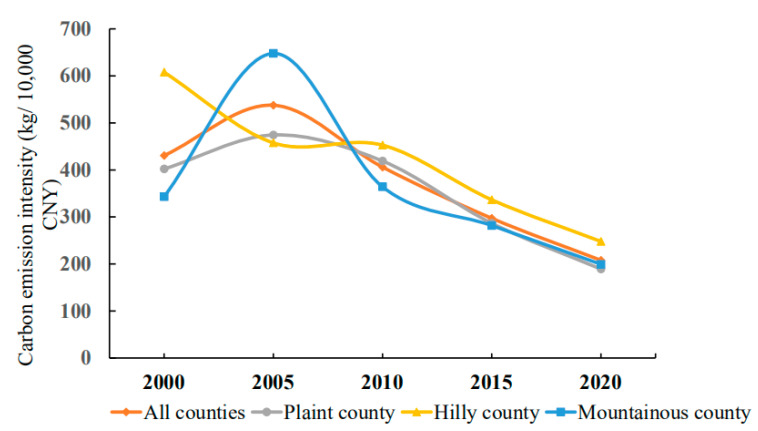
Topographic differences in carbon emission intensity in Hubei Province.

The carbon emission intensity may more accurately perform horizontal comparisons between areas since it is unaffected by the overall resource base and can indicate the low-carbon level of the planting industry in a certain region [8]. It can be seen from Figure 8 that from 2000 to 2020, the carbon emission intensity of counties, plain areas and mountainous areas in Hubei Province as a whole showed similar time series change characteristics, and the continuous decline state of “rising–falling” with 2005 as the dividing point (Figure 8), while the carbon emission intensity of hilly areas showed the characteristics of continuous decline. The traditional agricultural counties or the main grain producing areas in hilly and plain areas are the primary contributors of carbon emissions from the exploitation of cultivated land. This may be deduced from the general distribution. Among these are the highly intensive use of cultivated land resources, as well as the increased investment in high-carbon agricultural materials such as chemical fertilizer, agricultural film, pesticide and diesel oil, which ultimately result in a high total amount of carbon emissions from planting. 

### 4.3. Analysis on Decoupling Effect between Carbon Emission and Agricultural Economic Growth

Decoupling between carbon emissions and economic growth is examined in 72 counties of Hubei Province, and the features and emission reduction routes of distinct city types are identified using the Tapio decoupling elasticity index. The conventional Tapio model divides the decoupling status into eight categories. The most ideal state is strong decoupling. The ideal state is weak decoupling. The general state is recession decoupling. The less ideal states are weak negative decoupling and expanded negative decoupling. The worst state is strong negative decoupling. The unrelated state is the growth link and the recession link.

**Figure 9:**
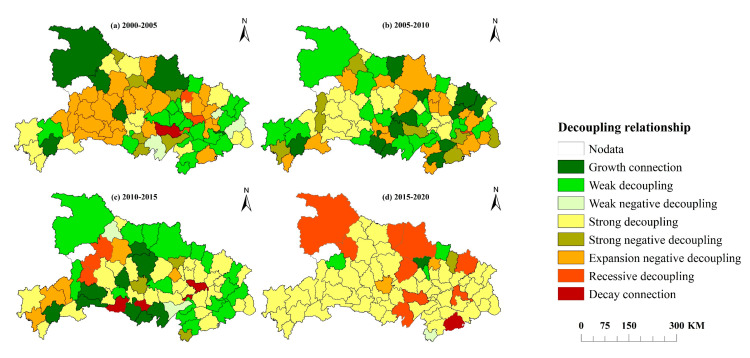
Decoupling characteristics between carbon emissions and agricultural economic growth in Hubei Province (Four stages).

This study uses ArcGIS 10.7 software and the natural breakpoint method to show the decoupling characteristics between the two in a visual way, as shown in Figure 9. Table A1, Table A2, Table A3 and Table A4 detail the decoupling elasticity and decoupling features of the decoupling connection between carbon emissions and agricultural economic development in 2000–2005, 2005–2010, 2010–2015, and 2015–2020. In Hubei Province, the decoupling features of carbon emissions and agricultural economic growth from 2000 to 2005, 2005 to 2010, 2010 to 2015 and 2015 to 2020 are significantly distinct. The majority of carbon emissions and agricultural economic growth in central and western Hubei were negatively decoupled from expansion from 2000 to 2005. Between 2005 and 2010, a significant number of counties and districts in western Hubei that were also in a condition of negative decoupling before the province′s development transformed into a state of robust decoupling. From 2010 to 2015, northern Hubei was in a weak decoupling state, and most areas of Jianghan Plain showed an obvious strong decoupling state. Between 2015 and 2020, most regions in Hubei Province showed strong decoupling between carbon emissions and agricultural economic growth, and a few regions showed recession decoupling.

Specifically, from 2005 to 2010, the number of counties and cities in the state of negative decoupling was 24, accounting for 33% of the total sample, i.e., accounting for a large proportion. This demonstrates that at the present moment, the agricultural economic expansion in Hubei Province prioritizes high input, high energy consumption and low output, resulting in economic growth at the expense of environmental pressure. The possible reason for this phenomenon is that Hubei Province, as a large grain-producing province, assumed important responsibility for agricultural safety during this period. Under the limited agricultural productivity and capital-scale investment, in order to ensure the stability of agricultural output, it had to use traditional factor investment on a large scale. For example, the abuse of pesticides and fertilizers is widespread, which makes agricultural non-point source pollution more serious. Therefore, at this time, agricultural economic growth and environmental pressure show a negative decoupling state.

From this result, it can be found that the number of counties and cities with strong decoupling status significantly increased from 2000 to 2020, which means that the environmental pressure of the planting industry in Hubei Province is gradually decreasing, and agricultural economic growth has begun to shift from dependence on cultivated land resources to a low-carbon development path. The target of carbon neutralization and carbon compliance and increased national focus on the treatment of agricultural non-point source pollution may be the cause, which has led Hubei Province to start paying greater attention to the green transformation of the agricultural industry. With the vigorous promotion of agricultural mechanization and ecological planting mode, carbon emissions began to decline on the premise of ensuring that the output level of cultivated land use was not reduced, so the decoupling effect between carbon emissions and agricultural economic growth was obvious.

### 4.4. Analysis of Carbon Emission Drivers

#### 4.4.1. Decomposition of the Driving Factors of Carbon Emission Based on the Overall Sample

**Figure 10:**
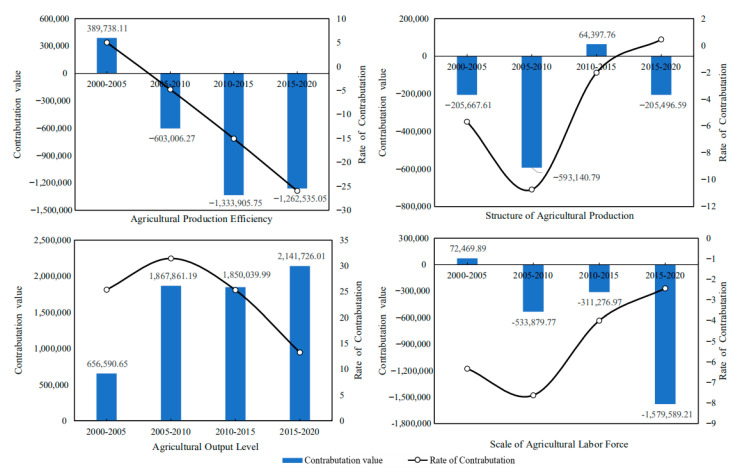
Contribution value and contribution rate of carbon emission drivers of cultivated land use in Hubei Province from 2000 to 2020 (including Agriculture Production Efficiency, Structure of Agriculture Production, Agriculture Output Level, Scale of Agricultural Labor Force).

On the basis of the LMDI model, the driving factors of carbon emission at the county level in Hubei Province are decomposed, and the contribution value and contribution rate of such driving factors as agricultural production efficiency, output level, production structure and labor scale to the carbon emission in Hubei Province from 2000 to 2020 can be obtained (Figure 10). In Figure 10, the positive value in the vertical axis indicates the effect of carbon emission increase, and the negative value indicates the effect of carbon emission reduction. The empirical results show that:

(1) Agricultural production efficiency is the main driving factor for the reduction in carbon emissions from cultivated land utilization in Hubei Province. From 2000 to 2020, the cumulative carbon emission reduction effect of agricultural production efficiency factors should reach 2.8097 million tons, with an average annual carbon emission reduction effect of approximately −702,400 tons. The contribution rate of carbon emission reduction is generally high and rising, indicating that the inhibitory effect of agricultural production efficiency on carbon emissions is increasing. It can be seen that, in the past two decades, the improvement of agricultural production efficiency has restrained the growth of planting carbon emissions in Hubei Province to a certain extent. Improving agricultural production efficiency will become an important measure to promote the low-carbon planting industry in Hubei Province.
(2) The agricultural production structure (the ratio of the total output value of planting industry to the total output value of agriculture, forestry, animal husbandry and fishery) is an important driving factor for the reduction in carbon emissions from planting industry in Hubei Province. From 2000 to 2020, the cumulative carbon emission reduction effect of agricultural production structure factors reached 939,900 tons, with an average annual carbon emission reduction effect of approximately −235,000 tons. The contribution rate of carbon emission reduction is low on the whole, and gradually shows a weakening trend. On the whole, the effect of a carbon emission increase is not obvious. This may be because the urbanization process in Hubei Province accelerated from 2000 to 2010, and a large number of rural residents transferred to cities, resulting in the reduction in rural cultivated land, and even the abandonment of cultivated land in some areas. From 2010 to 2020, in order to ensure grain production and national food security, and at the same time, thanks to the reform of rural land property rights system and the large-scale management of land brought about by land circulation, the production scale of a planting industry in Hubei province gradually expanded. It can be seen that it is increasingly difficult to reduce carbon emissions from farming by significantly adjusting the structure of agricultural production.
(3) The level of agricultural output is the main driving factor for the increase in carbon emissions from planting in Hubei Province. From 2000 to 2020, the cumulative carbon emission increase effect of agricultural output level factors reached 6.5162 million tons, with an average annual carbon emission increase effect of approximately 1.6291 million tons. The contribution rate of the carbon emission increase is on the high side as a whole. The increasing effect of agricultural output on carbon emissions is increasing from 2001 to 2010, but it tends to weaken after 2010. This may be because the improvement of the agricultural output level depends more on Agricultural Chemistry and agricultural mechanization, that is, it depends on a large number of inputs of high-carbon materials such as chemical fertilizers, agricultural films, pesticides, diesel oil, etc., which leads to the high carbon emissions of planting industry. However, under the guidance of agricultural green development, the carbon emission increase effect of the agricultural output level is gradually weakening.
(4) The scale of the agricultural labor force is one of the important driving factors for the reduction in carbon emissions from planting in Hubei Province. From 2005 to 2020, the cumulative carbon emission reduction effect of agricultural labor scale factor was approximately 2.3523 million tons, and the average annual carbon emission reduction effect was approximately 588,100 tons. The contribution rate of carbon emission reduction is generally high, but it is in a fluctuating trend of rising first and then declining. This may be because, with the promotion of urbanization and the progress of agricultural production technology, the rural labor surplus in Hubei Province is gradually transferred to the non-agricultural part, and the number of labors engaged in agriculture is gradually reduced, but the speed of non-agricultural labor is gradually slowing down. It can be seen that, although agricultural technological progress can partially replace labor, the dependence of a planting industry on labor still exists under the influence of a small-scale peasant economy and family farming methods.

#### 4.4.2. Decomposition of Carbon Emission Driving Factors of Cultivated Land Resource Utilization in Hubei Province Based on Terrain

Hubei Province has three landforms: plains, hills and mountains, and the natural resource endowment of agricultural production in different regions varies greatly, which may lead to differences in the driving factors of agricultural planting carbon emissions in different regions. Therefore, in order to obtain more abundant and accurate research findings, this paper further decomposes the driving factors of planting carbon emissions in Hubei Province based on the differences in topographic characteristics. It should be noted that the number of plain counties, hilly counties and mountainous counties in Hubei Province is inconsistent, so in order to eliminate the estimation errors that may be caused by the inconsistency in the number of counties with different terrain, the results here are the average effect of the number of carbon emission reductions or carbon emissions increases caused by different factors.

**Figure 11:**
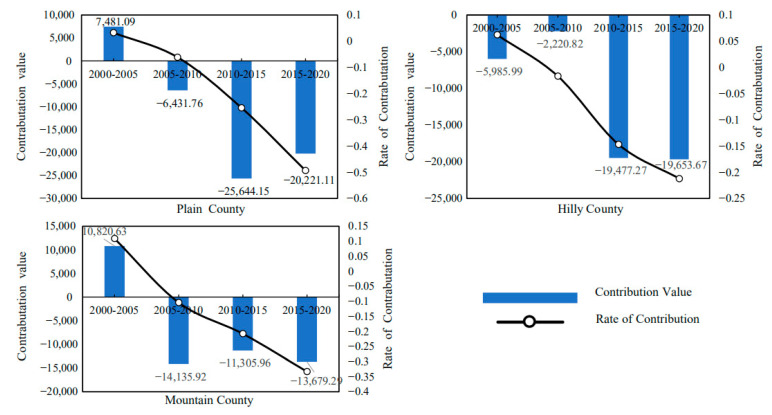
Topographic heterogeneity of agricultural production efficiency and emission reduction effect (including Plain County, Hilly County, Mountain County).

**Figure 12:**
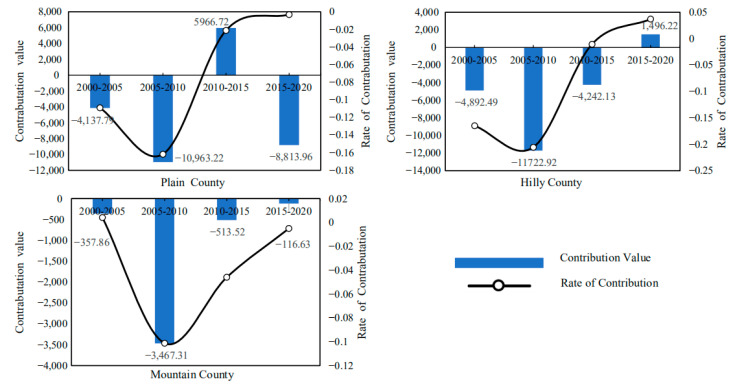
Topographic heterogeneity of emission reduction effect of agricultural production structure (including Plain County, Hilly County, Mountain County).

**Figure 13:**
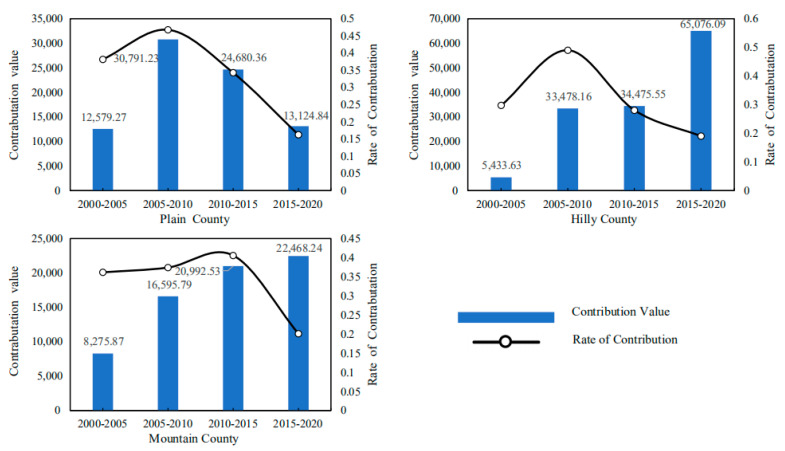
Topographic heterogeneity of agricultural output level increase and emission effect (including Plain County, Hilly County, Mountain County).

**Figure 14:**
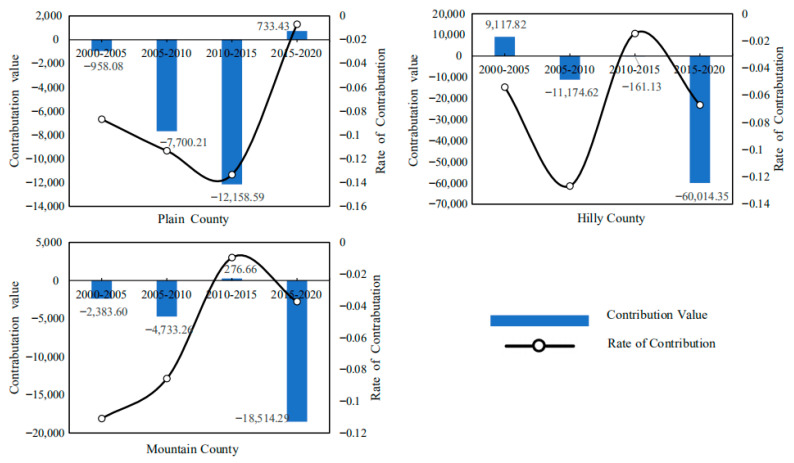
Topographic heterogeneity of emission reduction effect of agricultural labor scale (including Plain County, Hilly County, Mountain County).

Based on the decomposition of the LMDI model, the average contribution value and contribution rate of agricultural production efficiency, output level, production structure and labor force scale to the carbon emission of cultivated land use in Hubei Province from 2000 to 2020 can be obtained. The decomposition result shows:

(1) From the emission reduction effect of agricultural production efficiency, as shown in Figure 11, the cumulative carbon emission reduction in agricultural production efficiency factors in the plain area averaged 44,800 tons from 2000 to 2020. The cumulative carbon emission reduction in agricultural production efficiency factors in hilly areas is 47,300 tons on average. The cumulative carbon emission reduction in agricultural production efficiency factors in mountainous areas averages 28,300 tons. This shows that the emission reduction effect of agricultural production efficiency in plain and hilly areas is strong, while the carbon emission reduction effect of agricultural production efficiency in mountainous areas is relatively low. The possible reason is that the plain and hilly areas are conducive to the promotion and application of modern mechanized production, and the maturity of large-scale planting is higher, so the agricultural production efficiency is higher, while the carbon emission reduction effect of the agricultural production efficiency in mountain areas is limited and weakened due to the existence of topographic barriers. At the same time, it can be seen from the change of contribution rate curve that with the continuous development of modern agriculture, the carbon emission reduction effect brought by agricultural production efficiency factors is gradually increasing.
(2) From the perspective of the emission reduction effect of agricultural production structural factors, as shown in Figure 12, the cumulative carbon emission reduction in agricultural production structural factors in the plain area averaged approximately 17,900 tons from 2000 to 2020. The cumulative carbon emission reduction in agricultural production structure factors in hilly areas is approximately 19,300 tons on average. The cumulative carbon emission reduction in agricultural production structure factors in mountainous areas is approximately 4500 tons on average. The results show that the emission reduction effect of agricultural production structure factors in plain and hilly areas is greater. This may be because the planting industry in plain and hilly areas is large-scale, so its carbon emissions are significantly higher than those in mountain areas. At the same time, it can be seen from the change of the contribution rate curve that with the continuous advancement of agricultural modernization, the carbon emission reduction effect caused by the structural factors of agricultural production is gradually weakening.
(3) From the perspective of the increase in and emission effect of agricultural output level factors, as shown in Figure 13, the cumulative carbon increase in and emission of agricultural output level factors in the plain area averaged 81,200 tons from 2000 to 2020. The cumulative carbon emission increase in agricultural output level factors in hilly areas averaged 138,500 tons. The cumulative carbon emission increase in agricultural output level factors in mountainous areas is 68,300 tons on average. The above results show that the carbon emission increase effect brought by the agricultural output level in hilly areas is the strongest, which is significantly higher than that in plain and mountain areas. This may be because the hilly areas bear an important share of agricultural output, coupled with the relatively high level of agricultural labor input, which leads to a higher level of carbon emissions. However, it can be seen from the change of the contribution rate curve that the carbon emission increase effect caused by the level of agricultural output is gradually weakening.
(4) From the perspective of the emission reduction effect of the agricultural labor scale, as shown in Figure 14, the cumulative carbon emission reduction in agricultural labor scale factors in the plain area averaged 20,000 tons from 2000 to 2020. The cumulative carbon emission reduction in agricultural labor scale factors in hilly areas is approximately 62,200 tons on average. The cumulative carbon emission reduction in agricultural labor scale factors in mountainous areas is approximately 25,400 tons on average. The results show that the emission reduction effect of the agricultural labor force scale factor in the plain area is smaller. This may be related to the population and natural factors in the plain area. The agricultural production conditions in Jianghan Plain are good, the population is concentrated, and the agricultural production is dominated by small farmers. Farmers have a strong “cherish land” complex, and the per capita cultivated land is small, which is difficult to form the scale effect of cultivated land. At the same time, plain areas have convenient transportation and developed non-agricultural industries, so compared with mountainous and hilly areas, farmers in plain areas have a higher degree of part-time industrialization. It can be seen that Jianghan Plain should continue to accelerate the transfer of rural surplus labor, promote the large-scale management of agricultural land and then enhance the carbon emission reduction effect caused by the reduction in the scale of agricultural labor.

In general, in recent years, the improvement of planting production efficiency, the optimization of planting structure and the non-agricultural transfer of agricultural labor have had a positive impact on the carbon emission reduction in the planting industry in Hubei Province. The improvement of agricultural output is the most important factor in the increase in carbon emissions from planting in Hubei Province. It can be predicted that in the future, the level of agricultural output will still be the main driving factor to promote carbon emissions from planting production. In addition, there are obvious topographic differences in carbon emission reduction or carbon emission increase effects caused by various factors.

## 5. Discussion

### 5.1. Deficiency and Prospect

China, in the initial year of the 14th five-year plan, continued to promote high-quality development, and implemented “carbon peak” and “carbon neutral” actions, providing a strong driving force for sustainable economic and social development [62]. From 2000 to 2020, the IPCC carbon emission coefficient approach was used to assess carbon emissions in 72 counties in Hubei Province. The temporal changes and regional differences of carbon emissions are analyzed. The decoupling properties of carbon emission are disclosed using the Tapio model, and the LMDI model was used to further investigate the primary driving forces of carbon emission. Although this article discloses the spatiotemporal progression, decoupling status, and primary influencing elements of carbon emissions and economic growth to some extent, there is still potential for improvement and refinement [62].

The carbon emission coefficient of different crop types has certain differences, so the agricultural carbon emission and emission intensity in Hubei Province may also be related to the change of crop planting structure, but this paper does not discuss this problem [43]. The change of cultivated land use type is closely related to human activities [63]. In recent years, ecological protection activities such as returning cultivated land to forest, returning cultivated land to grassland and returning cultivated land to lake in Hubei Province have changed agricultural production, so the change of cultivated land use may also be an important factor in the change of agricultural carbon emissions, but this paper does not consider it.

In addition to the Logarithmic Mean Divisia Index (LMDI) utilized in this study, there are several approaches for analyzing the elements that influence carbon emissions, such as stochastic impacts by regression population, influence, and technology (STIRPAT), which was proposed by Ehrlich [64,65] and improved by Dietz [66]. It is widely used in the study of influencing factors of carbon emissions in various regions and industries [67,68]. In the follow-up research, the STIRPAT model can also be used to establish the decomposition model of influencing factors of land-use carbon emissions from the aspects of carbon emission intensity, carbon emission efficiency, energy intensity, economic scale, land scale, population scale, etc., so as to refine the driving force research [32,62].

In essence, the connection between carbon emissions and economic growth is a dialectical one between the coordinated expansion of the local economy, resources, and environment, with carbon emission reduction at its center [38]. The academic community has made fruitful achievements in the coupling and coordination of “energy–economy–environment” and “carbon emission reduction–economic growth–environmental protection” [29,35,69,70]. The research method also focuses on the panel data, using vector auto regression (VAR) [71,72], vector error correction model (VECM) [34,73,74,75] and global vector auto regression (GVAR) [73,74,75] are used to explore the deeper economic relationship between the two aspects. How to integrate existing methods and carry out detailed research on carbon emissions and economic growth from different perspectives also needs to be further explored [62].

### 5.2. Policy Enlightenment

The research findings, which are based on an analysis of the spatiotemporal characteristics and driving forces behind carbon emissions from cultivated land use in Hubei Province, can serve as a crucial guide for the sustainable development of agriculture in Hubei Province and the formulation of pertinent policies to support the high-quality development of agriculture. Accordingly, the following policy recommendations are put forward.

First, chemical fertilizer is the main carbon source of carbon emissions, and it is also key to promoting the low-carbon use of cultivated land resources [35]. Regions with a high carbon emission intensity of cultivated land resource utilization should formulate fertilizer reduction schemes according to local conditions [76]. The Agricultural Technology Extension Department of Hubei Province should promote the calculation of land fertility, and implement the quota supply of chemical fertilizer according to the calculation results [77]. Additionally, a progressive increase in the quantity of microbial fertilizer, bio organic fertilizer, and water-soluble fertilizer is recommended.

Second, according to the differences in carbon sources and carbon emissions among regions, differentiated emission reduction policies should be implemented to promote the overall reduction in carbon emissions in Hubei Province [51]. As the key area of grain production, plain areas have an irreplaceable position in ensuring national food security. Plain areas can reduce the use of pesticides, chemical fertilizers, agricultural films and other agricultural materials; increase the use of organic fertilizers; and develop low-carbon agriculture and ecological agriculture with high agricultural output value in accordance with local conditions, so as to reduce the growth rate and intensity of carbon emissions in the process of agriculture [78,79]. Hilly areas should make use of their own economic advantages, continue to optimize the allocation of agricultural means of production on the basis of maintaining low intensity; further reduce the proportion of traditional agriculture; and vigorously develop leisure agriculture, ecological agriculture and urban agriculture with high agricultural output value, so as to improve the versatility of agricultural production and make it develop in the direction of less carbon emission [80,81]. In addition, strict emission reduction tasks should be formulated for mountainous areas with a high intensity of agricultural carbon emissions. Starting from various sources of agricultural carbon emission, we should control emissions, increase investment in emission reduction funds, actively introduce agricultural carbon emission reduction technologies and finally reduce the intensity of agricultural carbon emission [34,62,67].

## 6. Conclusions

The primary concerns are the spatiotemporal properties and underlying causes of carbon emissions from cultivated land utilization. The system for measuring carbon emissions is set up in this study to satisfy the two main development requirements of high-quality agricultural growth and food security. The spatiotemporal characteristics and driving forces of carbon emissions in Hubei Province were explored, and the decoupling between carbon emissions and agricultural economic growth was investigated using county data from Hubei Province from 2000 to 2020. 

The following are the results: (1) Spatiotemporal variations in carbon emissions are evident. In terms of time, the magnitude of carbon emissions in Hubei Province is still substantial, and the “low-carbon” process of resource utilization on cultivated land is quite gradual. Geographically, the high-value region of the middle east in Hubei Province coexists with the low-value zone of the west, with apparent regional contrasts; (2) In Hubei Province, the decoupling effect between carbon emissions and agricultural economic growth is becoming increasingly apparent. The majority of counties are now in a strong decoupling condition, and the number of counties and cities in a negative decoupling state has dramatically dropped; and (3) In general, the efficiency of agricultural production, production structure, output level and labor scale all work together to raise carbon emissions. The most crucial motivator for reducing carbon emissions is agricultural production efficiency. According to the topography, the agricultural output level is a significant driving force to boost the carbon emission while the agricultural production efficiency is the main driving force to reduce it in the plains. The volume of agricultural production has a significant impact on the rise in carbon emissions in mountainous areas. The agricultural labor scale has a significant impact on the rise in carbon emissions in mountainous locations. These findings have some effects on lowering the magnitude of carbon emissions from cultivated land usage and attaining high-quality agricultural growth. 

This research examines the spatiotemporal aspects of carbon emissions from cultivated land utilization and its driving forces, as well as the decoupling effect between the amount of carbon emissions and agricultural economic growth, using county-level data. However, this work does not explicitly explore the effects of climate and lifestyle on carbon emissions, which is an area that requires future investigation.

## Figures and Tables

**Table 1:** Calculation formula and carbon emission coefficient of each carbon emission source.

Carbon Source	Formula	Carbon Source Input	Carbon Emission Coefficient	Reference
Chemical fertilizer	$C_{f}=G_{f}\times A$	$G_{f}—\mathrm{fertilizer}\;\mathrm{scalar}/\mathrm{kg}$	A=0.896	[44]
Pesticides	$C_{p}=G_{p}\times B$	$G_{p}—\mathrm{pesticide}\;\mathrm{usage}/\mathrm{kg}$	B=4.934	[45]
Agricultural film	$C_{m}=G_{m}\times D$	$G_{m}—\mathrm{agricultural}\;\mathrm{film}\;\mathrm{consumption}/\mathrm{kg}$	D=5.18	[46]
Agricultural machinery	$C_{e}=\left(A_{a}\times E\right)+\left(W_{e}\times F\right)$	$W_{e}—\mathrm{total}\;\mathrm{power}\;\mathrm{of}\;\mathrm{agricultural}\;\mathrm{machinery}/\mathrm{kW}$ $A_{a}—\mathrm{sown}\;\mathrm{area}\;\mathrm{of}\;\mathrm{crops}/{\mathrm{hm}}^{2}$	E=16.47 F=0.18	[47]
Irrigation	$C_{i}=A_{i}\times G$	$A_{i}—\mathrm{effective}\;\mathrm{irrigation}\;\mathrm{area}/{\mathrm{hm}}^{2}$	G=20.5	[48]
Ploughing	$C_{t}=A_{t}\times H$	$A_{t}—\mathrm{farming}\;\mathrm{sown}\;\mathrm{area}/{\mathrm{hm}}^{2}$	H=3.126	[49]

**Table 2:** Indicators and descriptions of eight decoupling statuses.

Decoupling Status		Δ*C/C*	Δ*G/G*	Elasticity *e*	Remarks
Negative decoupling	Expansion negative decoupling	>0	>0	*e* > 1.2	Both economic growth and carbon emissions have surged, with carbon emissions increasing at a higher pace than the economy.
	Strong negative decoupling	>0	<0	0 < *e*	Economic growth declines and carbon emissions rise.
	Weak negative decoupling	<0	<0	0 ≤ *e* < 0.8	Both economic growth and carbon emissions are increasing, with carbon emissions increasing at a higher pace.
Decoupling	Weak decoupling	>0	>0	0 ≤ *e* < 0.8	Carbon emissions increase along with economic expansion, which is accelerating.
	Strong decoupling	<0	>0	0 < *e*	Increasing economic expansion and decreasing carbon emissions.
	Recessive decoupling	<0	<0	*e* > 1.2	Both economic growth and carbon emissions have declined, with carbon emissions declining more quickly than economic growth.
Connect	Growth connection	>0	>0	0.8 ≤ *e* < 1.2	Both economic growth and carbon emissions are on the rise, and their rates of expansion are equal.
	Decay connection	<0	<0	0.8 ≤ *e* < 1.2	Carbon emissions have declined at the same pace as economic growth.

**Table 3:** Unit and source of each indicator.

Category	Unit	Data Sources
Agricultural output value	CNY	The Hubei Province Statistical Yearbook
Agricultural employees	10^4^ people	The Hubei Province Statistical Yearbook
Chemical fertilizer	10^4^ tons	Municipal Statistical Yearbook
Pesticides	Ton	Municipal Statistical Yearbook
Agricultural film	Ton	Municipal Statistical Yearbook
Total mechanical power	10^4^ kW	Hubei Rural Statistical Yearbook
Effective irrigation area	hm^2^	Hubei Rural Statistical Yearbook
Ploughing (sown area of crops)	hm^2^	Hubei Rural Statistical Yearbook

## Data Availability

Not applicable.

## Appendix A

**Table A1:** Decoupling characteristics of carbon emissions and agricultural economic growth from 2000 to 2005.

County	Ambient Pressure (Δ*C*/*C*)	Economic Growth (Δ*G*/*G*)	Decoupling Elasticity (*e*)	Decoupling Feature
Wuhan municipal District	−0.0843	−0.0122	6.9378	Recessive decoupling
Caidian District	0.0927	0.2049	0.4525	Weak decoupling
Jiangxia District	0.1036	0.1795	0.5771	Weak decoupling
Huangpi District	0.1372	0.0708	1.9384	Expansion negative decoupling
Xinzhou District	0.1158	0.0682	1.6975	Expansion negative decoupling
Shiyan City	0.2417	0.2655	0.9105	Growth connection
Huangshi municipal District	0.2807	0.2962	0.9478	Growth connection
Daye City	0.176	0.2881	0.6111	Weak decoupling
Yangxin County	0.1397	0.2946	0.474	Weak decoupling
Jingzhou municipal District	−0.0547	0.1058	−0.5166	Strong decoupling
Jiangling County	0.0572	−0.0344	−1.6598	Strong negative decoupling
Songzi City	0.0553	0.018	3.063	Expansion negative decoupling
Gongan County	0.0404	0.1419	0.2846	Weak decoupling
Cityshou City	0.1353	−0.0684	−1.978	Strong negative decoupling
Jianli County	−0.102	−0.2141	0.4764	Weak negative decoupling
Honghu City	0.7047	−0.2965	−2.3769	Strong negative decoupling
Yichang municipal District	0.8528	0.335	2.5455	Expansion negative decoupling
Yidu City	0.8664	0.0538	16.0957	Expansion negative decoupling
Zhijiang City	0.798	0.1518	5.2566	Expansion negative decoupling
Dangyang City	0.1526	0.1312	1.1628	Growth connection
Yuanan County	0.782	0.2826	2.7669	Expansion negative decoupling
Xingshan County	0.8593	0.0921	9.3303	Expansion negative decoupling
Zigui County	0.7131	0.0836	8.5326	Expansion negative decoupling
Changyang County	0.7081	0.2037	3.4763	Expansion negative decoupling
Wufeng County	0.6954	0.1535	4.5297	Expansion negative decoupling
Xiangyang municipal District	−0.178	0.6338	−0.2809	Strong decoupling
Laohekou City	0.2856	−0.0494	−5.7786	Strong negative decoupling
Zaoyang City	0.1109	0.0072	15.4605	Expansion negative decoupling
Yicheng City	0.0376	−0.1562	−0.2403	Strong negative decoupling
Nanzhang County	0.2179	0.1292	1.6863	Expansion negative decoupling
Gucheng County	−1.1319	0.0274	−41.3235	Strong decoupling
Baokang County	0.2633	0.258	1.0205	Growth connection
Ezhou City	0.6816	0.2498	2.7283	Expansion negative decoupling
Jingmen municipal District	0.4612	0.2631	1.7531	Expansion negative decoupling
Shayang County	−0.0154	0.0139	−1.108	Strong decoupling
Zhongxiang City	0.4044	0.0178	22.6867	Expansion negative decoupling
Jingshan County	0.0556	0.0225	2.4736	Expansion negative decoupling
Xiaogan municipal District	−0.2162	0.0689	−3.1391	Strong decoupling
Xiaochang County	−0.0146	−0.0028	5.2694	Recessive decoupling
Dawu County	0.0165	0.1569	0.1051	Weak decoupling
Anlu City	0.2503	−0.0081	−30.7713	Strong negative decoupling
Yunmeng County	0.0029	0.1843	0.0156	Weak decoupling
Yingcheng City	0.0084	0.1395	0.0601	Weak decoupling
Hanchuan City	0.041	0.1016	0.4036	Weak decoupling
Huanggang municipal District	0.1412	0.1051	1.3432	Expansion negative decoupling
Tuanfeng County	−0.0906	−0.1785	0.5074	Weak negative decoupling
Hongan County	0.7769	0.0527	14.7423	Expansion negative decoupling
Macheng City	−0.3949	0.2135	−1.8496	Strong decoupling
Luotian County	0.1073	0.1366	0.7851	Weak decoupling
Yingshan County	0.0202	0.0701	0.2883	Weak decoupling
Xishui County	0.0161	0.1798	0.0896	Weak decoupling
Qichun County	−0.0151	−0.0197	0.7671	Weak negative decoupling
Wuxue City	−0.117	0.1717	−0.6813	Strong decoupling
Huangmei County	−0.1906	0.2322	−0.8209	Strong decoupling
Xianan District	0.0277	0.1394	0.199	Weak decoupling
Jiayu County	0.2498	0.3809	0.6558	Weak decoupling
Chibi City	−0.0218	0.1784	−0.1223	Strong decoupling
Tongcheng County	0.0899	0.1927	0.4664	Weak decoupling
Chongyang County	0.1118	0.1739	0.6429	Weak decoupling
Tongshan County	0.1211	0.087	1.3929	Expansion negative decoupling
Zengdu District	0.3827	0.4723	0.8103	Growth connection
Enshi City	0.1643	0.232	0.7085	Weak decoupling
Lichuan City	0.4818	0.1692	2.8483	Expansion negative decoupling
Jianshi County	0.1698	0.1306	1.3005	Expansion negative decoupling
Badong County	−0.1239	0.2219	−0.5585	Strong decoupling
Xuanen County	0.1575	0.1823	0.864	Growth connection
Xianfeng County	−0.0557	0.1745	−0.3191	Strong decoupling
Laifeng County	−0.1222	0.1706	−0.7164	Strong decoupling
Hefeng County	−0.0102	0.0728	−0.1394	Strong decoupling
Xiantao City	−0.1084	−0.1137	0.9535	Decay connection
Tianmen City	0.0899	0.1303	0.6898	Weak decoupling
Qianjiang City	0.1524	0.0318	4.7877	Expansion negative decoupling

## Appendix B

**Table A2:** Decoupling characteristics of carbon emissions and agricultural economic growth from 2005 to 2010.

County	Ambient Pressure (Δ*C*/*C*)	Economic Growth (Δ*G*/*G*)	Decoupling Elasticity (*e*)	Decoupling Feature
Wuhan municipal District	−0.1526	0.0886	−1.7229	Strong decoupling
Caidian District	−0.0283	0.151	−0.1873	Strong decoupling
Jiangxia District	−0.1502	0.1435	−1.0465	Strong decoupling
Huangpi District	0.2876	0.2273	1.2653	Expansion negative decoupling
Xinzhou District	0.0386	0.0344	1.1214	Growth connection
Shiyan City	0.143	0.2889	0.4949	Weak decoupling
Huangshi municipal District	−0.7571	−0.409	1.8513	Recessive decoupling
Daye City	0.1453	−0.0378	−3.8467	Strong negative decoupling
Yangxin County	0.2463	0.0764	3.2218	Expansion negative decoupling
Jingzhou municipal District	0.3658	0.2553	1.4328	Expansion negative decoupling
Jiangling County	0.2508	0.1082	2.3174	Expansion negative decoupling
Songzi City	0.0673	0.1477	0.4557	Weak decoupling
Gongan County	0.2193	0.2054	1.0679	Growth connection
Cityshou City	0.1534	0.1775	0.8641	Growth connection
Jianli County	0.2507	0.328	0.7645	Weak decoupling
Honghu City	0.1799	0.2413	0.7456	Weak decoupling
Yichang municipal District	−1.3372	0.1826	−7.3229	Strong decoupling
Yidu City	−4.2845	0.43	−9.9642	Strong decoupling
Zhijiang City	−1.9146	0.2948	−6.4937	Strong decoupling
Dangyang City	0.0986	0.3209	0.3071	Weak decoupling
Yuanan County	−3.0973	0.2628	−11.7879	Strong decoupling
Xingshan County	−5.7343	0.3004	−19.0873	Strong decoupling
Zigui County	−0.7954	0.2751	−2.8911	Strong decoupling
Changyang County	−1.1123	0.1516	−7.3352	Strong decoupling
Wufeng County	−0.6602	0.3317	−1.9906	Strong decoupling
Xiangyang municipal District	0.0926	0.2386	0.3882	Weak decoupling
Laohekou City	−0.1962	0.0384	−5.1067	Strong decoupling
Zaoyang City	0.0985	0.0855	1.1519	Growth connection
Yicheng City	0.228	0.1071	2.1279	Expansion negative decoupling
Nanzhang County	0.1999	0.2952	0.6771	Weak decoupling
Gucheng County	0.5189	−0.0896	−5.7923	Strong negative decoupling
Baokang County	0.3032	0.2274	1.3333	Expansion negative decoupling
Ezhou City	0.0325	0.2083	0.156	Weak decoupling
Jingmen municipal District	−0.0409	0.0543	−0.7528	Strong decoupling
Shayang County	0.1139	0.1724	0.6606	Weak decoupling
Zhongxiang City	−0.0647	0.1175	−0.5504	Strong decoupling
Jingshan County	0.2235	0.1334	1.6752	Expansion negative decoupling
Xiaogan municipal District	0.438	0.1988	2.2039	Expansion negative decoupling
Xiaochang County	0.4383	0.1644	2.6662	Expansion negative decoupling
Dawu County	0.1154	0.2335	0.4943	Weak decoupling
Anlu City	0.1047	0.09	1.1633	Growth connection
Yunmeng County	0.0296	0.1664	0.1777	Weak decoupling
Yingcheng City	−0.0341	0.0546	−0.6246	Strong decoupling
Hanchuan City	0.082	0.1331	0.6161	Weak decoupling
Huanggang municipal District	0.1913	0.3011	0.6353	Weak decoupling
Tuanfeng County	0.335	0.1731	1.935	Expansion negative decoupling
Hongan County	−0.0788	0.1302	−0.6051	Strong decoupling
Macheng City	0.1783	0.1685	1.058	Growth connection
Luotian County	0.2663	0.2687	0.9908	Growth connection
Yingshan County	−0.2629	0.4381	−0.6	Strong decoupling
Xishui County	0.1909	0.1543	1.2372	Expansion negative decoupling
Qichun County	0.154	0.3338	0.4615	Weak decoupling
Wuxue City	0.3518	0.1764	1.9947	Expansion negative decoupling
Huangmei County	0.3459	−0.0025	−140.1991	Strong negative decoupling
Xianan District	0.1196	0.1053	1.1363	Growth connection
Jiayu County	0.0752	0.1488	0.5052	Weak decoupling
Chibi City	0.105	0.0688	1.5252	Expansion negative decoupling
Tongcheng County	0.3166	0.1536	2.0611	Expansion negative decoupling
Chongyang County	0.1512	0.1473	1.0268	Growth connection
Tongshan County	0.1024	−0.0254	−4.0291	Strong negative decoupling
Zengdu District	0.2182	0.1632	1.3364	Expansion negative decoupling
Enshi City	0.0594	0.1109	0.5357	Weak decoupling
Lichuan City	0.3783	0.525	0.7205	Weak decoupling
Jianshi County	−0.2236	0.1669	−1.3392	Strong decoupling
Badong County	0.042	−0.5984	−0.0702	Strong negative decoupling
Xuanen County	0.2369	0.2271	1.0431	Growth connection
Xianfeng County	0.3238	−0.0323	−10.0232	Strong negative decoupling
Laifeng County	0.3037	0.0534	5.6871	Expansion negative decoupling
Hefeng County	0.7357	0.1659	4.4341	Expansion negative decoupling
Xiantao City	0.0519	−0.0128	−4.0425	Strong negative decoupling
Tianmen City	0.1197	0.1326	0.9029	Growth connection
Qianjiang City	0.2067	0.1906	1.0844	Growth connection

## Appendix C

**Table A3:** Decoupling characteristics of carbon emissions and agricultural economic growth from 2010 to 2015.

County	Ambient Pressure (Δ*C*/*C*)	Economic Growth (Δ*G*/*G*)	Decoupling Elasticity (*e*)	Decoupling Feature
Wuhan municipal District	−0.5339	−0.4884	1.093	Decay connection
Caidian District	−0.1156	0.4699	−0.2461	Strong decoupling
Jiangxia District	−0.2188	0.5378	−0.4069	Strong decoupling
Huangpi District	−0.4588	0.5155	−0.89	Strong decoupling
Xinzhou District	−0.009	0.5385	−0.0168	Strong decoupling
Shiyan City	0.2154	0.4964	0.4339	Weak decoupling
Huangshi municipal District	0.1226	0.414	0.2962	Weak decoupling
Daye City	0.0484	0.3872	0.1249	Weak decoupling
Yangxin County	0.1905	0.3833	0.497	Weak decoupling
Jingzhou municipal District	−0.1842	0.188	−0.9795	Strong decoupling
Gongan County	0.5958	0.6936	0.8589	Growth connection
Jianli County	0.5465	0.667	0.8193	Growth connection
Jiangling County	−1.0539	−0.9733	1.0828	Decay connection
Cityshou City	0.1928	0.1824	1.0568	Growth connection
Honghu City	−0.2386	−0.509	0.4687	Weak negative decoupling
Songzi City	−0.0661	−0.064	1.0316	Decay connection
Yichang municipal District	−0.1232	0.1466	−0.8403	Strong decoupling
Yidu City	−0.4627	0.6654	−0.6953	Strong decoupling
Zhijiang City	0.6522	0.8224	0.793	Weak decoupling
Dangyang City	0.7461	0.784	0.9517	Growth connection
Yuanan County	−0.9347	0.1378	−6.7813	Strong decoupling
Xingshan County	−7.3048	−1.6579	4.4061	Recessive decoupling
Zigui County	−1.3684	−0.7351	1.8617	Recessive decoupling
Changyang County	0.6436	0.6272	1.0261	Growth connection
Wufeng County	0.6947	0.6447	1.0776	Growth connection
Xiangyang municipal District	0.4481	0.7732	0.5795	Weak decoupling
Nanzhang County	0.4193	0.1933	2.1686	Expansion negative decoupling
Gucheng County	−0.4905	−1.6472	0.2978	Weak negative decoupling
Baokang County	−2.5222	−0.4299	5.8669	Recessive decoupling
Laohekou City	−0.2298	0.3917	−0.5866	Strong decoupling
Zaoyang City	0.6449	0.8369	0.7706	Weak decoupling
Yicheng City	0.7069	0.731	0.967	Growth connection
Ezhou City	−0.2772	0.2204	−1.2576	Strong decoupling
Jingmen municipal District	−0.3529	0.139	−2.5389	Strong decoupling
Shayang County	−0.0847	0.1347	−0.6288	Strong decoupling
Zhongxiang City	0.2189	−0.1305	−1.6772	Strong negative decoupling
Jingshan County	0.4992	0.4239	1.1775	Growth connection
Xiaogan municipal District	−0.0562	0.1351	−0.4158	Strong decoupling
Xiaochang County	−0.2041	0.4056	−0.5032	Strong decoupling
Dawu County	0.1644	0.4328	0.3799	Weak decoupling
Yunmeng County	−1.3063	0.4563	−2.8625	Strong decoupling
Yingcheng City	0.4646	0.3503	1.3263	Expansion negative decoupling
Anlu City	0.3423	−0.2159	−1.5856	Strong negative decoupling
Hanchuan City	−0.0732	0.316	−0.2317	Strong decoupling
Huanggang municipal District	−0.0777	0.2818	−0.2757	Strong decoupling
Tuanfeng County	−0.0613	0.2184	−0.2809	Strong decoupling
Hongan County	0.0917	0.1352	0.6781	Weak decoupling
Luotian County	0.2587	0.374	0.6918	Weak decoupling
Yingshan County	−0.1416	0.2504	−0.5653	Strong decoupling
Xishui County	0.2072	0.2705	0.7658	Weak decoupling
Qichun County	0.1109	0.3681	0.3013	Weak decoupling
Huangmei County	−1.1574	0.3305	−3.502	Strong decoupling
Macheng City	−0.0166	0.2657	−0.0624	Strong decoupling
Wuxue City	−0.0302	0.2776	−0.1089	Strong decoupling
Xianan District	−0.0646	0.2497	−0.2588	Strong decoupling
Jiayu County	0.1772	0.4076	0.4347	Weak decoupling
Tongcheng County	0.0936	−0.0475	−1.971	Strong negative decoupling
Chongyang County	0.1918	0.4601	0.4168	Weak decoupling
Tongshan County	−1.0363	0.1024	−10.1235	Strong decoupling
Chibi City	0.6122	0.8094	0.7564	Weak decoupling
Zengdu District	0.0576	0.2438	0.2363	Weak decoupling
Enshi City	0.1106	0.086	1.2856	Expansion negative decoupling
Lichuan City	−0.1066	0.1749	−0.6099	Strong decoupling
Jianshi County	0.3877	0.083	4.6724	Expansion negative decoupling
Badong County	0.1272	0.1767	0.72	Weak decoupling
Xuanen County	0.2747	0.2323	1.1824	Growth connection
Xianfeng County	0.3409	0.1406	2.4252	Expansion negative decoupling
Laifeng County	−0.1985	0.2255	−0.8802	Strong decoupling
Hefeng County	−0.0326	0.1907	−0.171	Strong decoupling
Xiantao City	−0.0389	0.2662	−0.1461	Strong decoupling
Tianmen City	0.0086	0.1957	0.0437	Weak decoupling
Qianjiang City	−0.4066	0.1712	−2.3749	Strong decoupling

## Appendix D

**Table A4:** Decoupling characteristics of carbon emissions and agricultural economic growth from 2015 to 2020.

County	Ambient Pressure (Δ*C*/*C*)	Economic Growth (Δ*G*/*G*)	Decoupling Elasticity (*e*)	Decoupling Feature
Wuhan municipal District	−0.6522	0.2188	−2.9808	Strong decoupling
Caidian District	−0.1652	0.1467	−1.1262	Strong decoupling
Jiangxia District	−0.1597	0.1597	−0.9999	Strong decoupling
Huangpi District	−0.1646	0.2081	−0.7909	Strong decoupling
Xinzhou District	−0.3098	0.0444	−6.9807	Strong decoupling
Shiyan City	−0.6745	−0.0361	18.6657	Recessive decoupling
Huangshi municipal District	−1.3918	−0.0666	20.8921	Recessive decoupling
Daye City	−0.1962	0.1416	−1.3859	Strong decoupling
Yangxin County	−0.1822	0.1138	−1.6004	Strong decoupling
Jingzhou municipal District	−0.2322	0.1894	−1.2258	Strong decoupling
Gongan County	−0.6422	0.0921	−6.9759	Strong decoupling
Jianli County	−0.3238	−0.0601	5.3846	Recessive decoupling
Jiangling County	−0.1529	0.2184	−0.6998	Strong decoupling
Cityshou City	−0.4163	0.1769	−2.3529	Strong decoupling
Honghu City	−0.6196	0.018	−34.4189	Strong decoupling
Songzi City	−0.4818	0.1973	−2.4421	Strong decoupling
Yichang municipal District	−2.4889	0.06	−41.4827	Strong decoupling
Yidu City	−0.2088	0.2591	−0.8058	Strong decoupling
Zhijiang City	−0.1603	0.213	−0.7525	Strong decoupling
Dangyang City	−0.1886	0.105	−1.7967	Strong decoupling
Yuanan County	−0.1825	0.1627	−1.1214	Strong decoupling
Xingshan County	0.0239	0.1749	0.1369	Weak decoupling
Zigui County	−0.2043	0.3213	−0.6356	Strong decoupling
Changyang County	−0.4715	0.3977	−1.1856	Strong decoupling
Wufeng County	−0.2637	0.146	−1.8058	Strong decoupling
Xiangyang municipal District	−0.5986	0.0976	−6.1303	Strong decoupling
Nanzhang County	−0.6656	0.1697	−3.9225	Strong decoupling
Gucheng County	−1.1968	0.2959	−4.0441	Strong decoupling
Baokang County	−0.8771	−0.0282	31.1522	Recessive decoupling
Laohekou City	−0.075	0.1464	−0.5122	Strong decoupling
Zaoyang City	−0.6873	−0.0465	14.7856	Recessive decoupling
Yicheng City	−0.1009	0.0521	−1.9373	Strong decoupling
Ezhou City	−0.2783	−0.1318	2.111	Recessive decoupling
Jingmen municipal District	−0.1391	0.1877	−0.7413	Strong decoupling
Shayang County	0.4212	0.1668	2.5257	Expansion negative decoupling
Zhongxiang City	−0.1293	0.2963	−0.4363	Strong decoupling
Jingshan County	−0.565	−0.2505	2.2558	Recessive decoupling
Xiaogan municipal District	−3.1192	0.1137	−27.4265	Strong decoupling
Xiaochang County	0.3192	0.1049	3.0424	Expansion negative decoupling
Dawu County	0.0802	0.1022	0.7842	Weak decoupling
Yunmeng County	0.0881	0.1499	0.5879	Weak decoupling
Yingcheng City	−0.2743	0.0804	−3.4106	Strong decoupling
Anlu City	0.2005	0.2303	0.8705	Growth connection
Hanchuan City	−0.0321	0.0648	−0.4955	Strong decoupling
Huanggang municipal District	−0.3401	0.1017	−3.3439	Strong decoupling
Tuanfeng County	−0.3132	0.0587	−5.339	Strong decoupling
Hongan County	0.0937	−0.1019	−0.9198	Strong negative decoupling
Luotian County	−0.0912	0.0008	−119.0521	Strong decoupling
Yingshan County	−0.2446	0.1843	−1.3272	Strong decoupling
Xishui County	−0.0534	0.1363	−0.3915	Strong decoupling
Qichun County	−0.1511	0.0043	−34.7358	Strong decoupling
Huangmei County	−0.5843	0.024	−24.3088	Strong decoupling
Macheng City	−0.0603	−0.0058	10.3777	Recessive decoupling
Wuxue City	−0.7615	0.0365	−20.8813	Strong decoupling
Xianan District	−0.0548	0.1149	−0.4771	Strong decoupling
Jiayu County	−0.081	0.1589	−0.51	Strong decoupling
Tongcheng County	−0.006	−0.0937	0.0641	Weak negative decoupling
Chongyang County	−0.3339	0.1222	−2.7332	Strong decoupling
Tongshan County	−0.1354	−0.1148	1.1796	Decay connection
Chibi City	−0.0205	0.1751	−0.1171	Strong decoupling
Zengdu District	−0.127	−0.0067	19.0422	Recessive decoupling
Enshi City	−0.1962	0.1234	−1.5896	Strong decoupling
Lichuan City	−0.1743	0.0153	−11.4194	Strong decoupling
Jianshi County	−0.4292	0.277	−1.5496	Strong decoupling
Badong County	−0.0479	0.2327	−0.2059	Strong decoupling
Xuanen County	−0.0886	0.1906	−0.4647	Strong decoupling
Xianfeng County	−0.0185	0.36	−0.0515	Strong decoupling
Laifeng County	−0.1387	0.2822	−0.4916	Strong decoupling
Hefeng County	−0.3658	0.1442	−2.5372	Strong decoupling
Xiantao City	−0.498	−0.0641	7.7734	Recessive decoupling
Tianmen City	−0.0918	0.1609	−0.5704	Strong decoupling
Qianjiang City	−0.3867	0.0777	−4.9777	Strong decoupling
